# Measuring Cross-Cultural Supernatural Beliefs with Self- and Peer-Reports

**DOI:** 10.1371/journal.pone.0164291

**Published:** 2016-10-19

**Authors:** Matthias Bluemke, Jonathan Jong, Dennis Grevenstein, Igor Mikloušić, Jamin Halberstadt

**Affiliations:** 1 Psychological Institute, Ruprecht-Karls-Universitän Heidelberg, Heidelberg, Germany; 2 GESIS—Leibniz Institute for the Social Sciences, Mannheim, Germany; 3 Centre for Research in Psychology, Behaviour, & Achievement, Coventry University, Coventry, United Kingdom; 4 Institute of Cognitive and Evolutionary Anthropology, University of Oxford, Oxford, United Kingdom; 5 Institute of Social Sciences Ivo Pilar, Zagreb, Croatia; 6 Department of Psychology, University of Otago, Dunedin, New Zealand; Tilburg University, NETHERLANDS

## Abstract

Despite claims about the universality of religious belief, whether religiosity scales have the same meaning when administered inter-subjectively–or translated and applied cross-culturally–is currently unknown. Using the recent “Supernatural Belief Scale” (SBS), we present a primer on how to verify the strong assumptions of measurement invariance required in research on religion. A comparison of two independent samples, Croatians and New Zealanders, showed that, despite a sophisticated psychometric model, measurement invariance could be demonstrated for the SBS except for two noninvariant intercepts. We present a new approach for inspecting measurement invariance across self- and peer-reports as two dependent samples. Although supernatural beliefs may be hard to observe in others, the measurement model was fully invariant for Croatians and their nominated peers. The results not only establish, for the first time, a valid measure of religious supernatural belief across two groups of different language and culture, but also demonstrate a general invariance test for distinguishable dyad members nested within the same targets. More effort needs to be made to design and validate cross-culturally applicable measures of religiosity.

## Introduction

There has, in the past two decades, been burgeoning interest in the scientific study of religion as a psychological universal. In this time, many testable theories about the cognitive and evolutionary underpinnings of religious belief and behavior have been proposed. It is argued, for example, that the belief in supernatural agents emerged out of a hypersensitive tendency to detect agents in our surrounding environments [1, 2] and our ability to impute rich mental states to agents [3]. These two basic social cognitive processes are thought to lead to teleofunctional reasoning [4, 5]—the promiscuous tendency to perceive purpose in the world—which is itself a building block of religious belief (e.g., creationist ideas, beliefs about fate). There are also motivational accounts of religious belief, such that some basic, pancultural fears—of randomness [6] or death [7]—explain our attraction toward religion.

Whatever the substance of these theories of religion, they share in common the assumption of cross-cultural generalizability. That is, these are not theories about the belief in specific gods, but in gods in general. Unfortunately, the empirical progress in this nascent cognitive science of religion has lagged behind its theoretical fecundity, not least because there are limited tools available for reliably measuring cross-cultural religious beliefs. Most of the available measures are either deliberately or inadvertently too culturally specific for widespread cross-cultural use [8, 9]. Furthermore, the most commonly used measures of religiosity tend to be measures of religious participation (e.g., religious service attendance) or religious orientation—intrinsic, extrinsic, and quest, for example [10]—rather than measures of individuals’ tendencies to believe in supernatural entities and events. It was to address this methodological gap that Jong, Bluemke, and Halberstadt [11] designed the Supernatural Belief Scale (SBS), the items of which were derived from consulting anthropological texts to ascertain cross-culturally recurring supernatural beliefs.

The SBS is an attempt to measure an important *cognitive* aspect of religion: the belief in supernatural entities and events. Religion itself is a multi-faceted phenomenon. Indeed, scholars have long argued [12–15], the term “religion” unhelpfully lumps together a variety of related phenomena, including beliefs, behaviours, experiences, and social identities.

This recognition has led to the proliferation of multidimensional measures of religion, which attempt to capture multiple facets of religion within a single scale. Such measures are often used uncritically by researchers who treat them as unidimensional measures of general religiosity [16]. In contrast, the SBS specifically targets supernatural belief; it may therefore be used alongside measures of spiritual well-being or religious orientation, identity, behavior, and experience, but should not be confused for a proxy for those aspects of religiosity.

While the SBS may still be more suitable for some religious or cultural contexts (e.g., those that include worship of high gods) than others, those contexts include the vast majority of the world’s religious people. Also the SBS is a direct measure of a key theoretical construct in lay conceptions of “religiosity” and in recent cognitive research of religion, namely the belief in supernatural entities, which also occurs outside of organized forms of religion [1, 17–19]. Notwithstanding more general supernatural beliefs, the SBS represents an essentially unidimensional measure of supernatural beliefs prevalent in the majority of religious world-views. The assessment is conceptually independent of religious self-categorization and uncontaminated with religious practices or individual experiences that one may or may not have.

### Measuring Trait Supernatural Belief

As alluded to above, there are a number of cross-culturally recurring religious themes, and the SBS includes the most important, anthropologically constant themes. The scale consists of ten statements (S1 Table) to which respondents indicate their agreement on Likert scales, with the midpoint indicating explicit uncertainty or agnosticism. The items pertain to the belief in a high god (item 1), the belief in good and evil spiritual powers (e.g., angels, demons; items 2 to 4), the belief in a spiritual essence in human beings (e.g., soul; item 5), the belief in a spiritual realm (item 6), the belief in positive and negative conceptions of the afterlife (e.g., heaven, hell; items 7 and 8), the belief in inexplicable events (e.g., miracles; item 9), and the belief in spiritual intermediaries (e.g., prophets; item 10).

As supernatural beliefs come in specific forms, their core themes need to be assessable across different contexts. The SBS was explicitly designed to be amenable to translation and cross-cultural adaptation. For example, while the word “soul” was used in the original version, for use among urban New Zealanders, the word “atta” or “atman” may be used instead in contexts where Pali- or Sanskrit-based religious traditions (e.g., Hinduism, Buddhism) are more culturally influential. Similarly, while “prophet” was originally used, “shaman” or “medium” may be preferable in other cultural contexts. Furthermore, single items may be dropped from the scale in cultures where certain otherwise commonly recurring concepts are conspicuously absent, without endangering the interpretation of the remainder of the construct (presuming that psychometric validity still holds). The SBS provides a basic template for a flexible and cross-culturally adaptable measure of an individual’s tendency to believe in (religious) supernatural beliefs, which contemporary evolutionary and cognitive scientific studies on religion take as their primary explanandum.

Hypothesizing a unidimensional structure for supernatural belief, Jong and colleagues [11] tested eight alternative measurements models (Models M1-M8; see S1–S8 Figs) via a rigorous confirmatory factor analytic approach (CFA) on the SBS. Across two independent samples as well as the combined sample, the SBS was shown to have an essentially unidimensional structure, qualified by a factor for items with negative valence (e.g., evil spirits, negative conceptions of afterlife), and five content facets (Model M5). The accepted model extracted on average 81% of the variance from the test items, indicating reliable inter-individual differences of test scores [20]. The optimally weighted item combination yielded a supernatural belief factor at the latent level that was assessed almost perfectly reliably, Ω_w_ = .95 [21]. Furthermore, average SBS scores predicted importance of religion to identity (*r* = .54), and religious service attendance (ρ = .60). The SBS was also shown to be useful for testing hypotheses about the relationship between death anxiety and religious belief [22].

### What are Measurement Invariance (MI) Tests?

The SBS provides a reliable, valid, and useful measure of religiosity in the cultural context in which it was developed. But to be useful for testing hypotheses about cross-cultural universals, any religiosity measure has to function equivalently across diverse cultures and languages [23]. Yet it is common practice in research on religion to simply apply instruments developed in one culture to another. In large-scale cross-cultural surveys applications of single item measures of religiosity are common, but they prevent the investigation of equivalent functioning across groups. Thus, when comparing scale scores, a crucial assumption is that a construct is measured in the same way in all of the compared groups. Also, when other informants from the same language are examined to establish convergent validity by means of self-other correlations, self- and peer-ratings must be assumed to reflect the same construct [24]. The assumption formally presupposes measurement invariance, which should be rigorously tested [25].

Measurement invariance is the attribute that an instrument (e.g., SBS) measures the same construct to the same extent in two different groups or data-sets (e.g., New Zealand vs. Croatia; self- vs. peer-reports). More formally, measurement invariance, or measurement equivalence, can be defined as the absence of group-based bias: Given an individual’s true score, the group membership should not affect the probability of obtaining a specific observed score [25–27]. Hence, the psychometric properties, which relate the observed variables to the latent variable, need to be similar across groups. Otherwise, meaningful comparisons between groups are severely hampered, if not impossible [25, 28–30]. With a few notable exceptions in the field of religion and spirituality, measurement invariance is hardly ever examined; more often there are attempts at replicating number of factors and indicator-factor patterns, and inspecting item means and standard deviations. If measurement invariance is ever tested, a typical outcome is limited cross-cultural generalizability [31, 32].

The current research focuses on the assumption of measurement invariance when assessing supernatural belief: (a) We test the translatability and cross-cultural applicability of the SBS, taking two “Western” countries–one from the Northern, one from the Southern Hemisphere–as a starting point; (b) we provide stringent evidence for its convergent validity in terms of self-peer-agreement; to do so (c) we present a new measurement invariance testing strategy that accommodates the dependent nature of self- and peer-data. Measurement invariance is most frequently tested by multiple-group confirmatory factor analysis (MGCFA), which assumes the independence of the groups being compared. Yet, whenever researchers use peer-reports to validate self-reports, the individual is no longer the sampling unit. This is a problem for the statistical analysis. Given the violation of the independence assumption, biased parameters and standard errors will result. We suggest measurement invariance tests that account for the statistical dependency of such dyadic data [33]. We term this approach *dependent-group confirmatory factor analysis* (DGCFA). It can be applied to validation approaches of any psychological construct based on self-other data that are nested within the same targets, including any behavioral measures with a cross-informant component, or other forms of dependency between whole groups.

Establishing measurement invariance requires a sequential test of hierarchical model specifications, depending in part on the goals of the study, and inspecting model fit [34, 35]. Which kind of research questions can be asked depends on the specific levels of measurement invariance achieved (cf. Table 1). One naming scheme for the degrees of measurement invariance is *weak*, *strong*, *strict factorial invariance*, after which follows the inspection of *full invariance* or *structural invariance* [25]. We use the naming scheme that describes the increasing equality constraints within the framework of confirmatory factor analysis [36, 37], resulting in four MI-levels: (a) *Configural invariance*: The least stringent test examines if the same number of factors and the items that load on these factors apply to both groups. There are no equivalency constraints in this model [38]. The configural invariance model serves as a baseline model for further MI-tests. (b) *Metric invariance*: Equal factor loadings across groups imply that the underlying scale is the same across groups, so participants use a common metric. Metric invariance is both necessary and sufficient for comparing factor correlations across groups. Without it, groups do not share an understanding of the construct [28]. (c) *Scalar invariance*: Adding equal regression intercepts across groups (also known as item thresholds) tests for equivalent item difficulties, so that group bias does not impact on factor means at the latent level. Legitimate comparisons of factor means across groups requires scalar invariance, because it ensures fairness and equity [34]. As Chen writes, “Otherwise, it is not certain whether group differences on factor means are attributable to valid cultural differences or to measurement artifacts” [28] (p.1006). (d) *Uniqueness invariance*: Finally, equal residual variances across groups imply that the item uniqueness terms are equivalent. This level ensures that the unexplained error variance of each item is the same between groups, which is required to compare observed scores (e.g., item or scale means) across groups. Otherwise, the means of residuals might differ because the errors represent unmodeled systematic variance, but differently so across groups. Alternatively, different item intercepts in the regressions might have been masked by unequal residual variance [39–42]. This MI-level must also hold if achieving equally reliable measurement across groups is an objective (this latter aspect additionally calls for equal factor variances) [37].

**Table 1:** Levels of Measurement Invariance: Equality Constraints, Model Identification, and Permissible Comparisons.

	Equality Constraint Across Groups	Model Identification	Implications	Permissible Comparison Across Groups
*Measurement Invariance*				
Configural	None (except for identical factor structure)	Covariance and mean structures identified like any SEM	Similar, but not identical constructs (equal dimensionality, factor form)	Further MI-levels
Metric	Factor loadings	Factor variances in the reference group = 1	Units of measurement (scaling)	(Co-)Variances of latent variables between or within scales
Scalar	Item intercepts	Factor means in the reference group = 0	Item difficulty (bias)	Latent factor means
Uniqueness	Residual variances	-	Amount of unique variance (including error variance)	Manifest item and scale properties (*M*, *SD*, reliability) including correlations
*Structural Invariance*				
Factor variances	Factor variances	-	-	Amount of variability, reliability
Factor covariances	Factor covariances	-	-	Cross-culturally replicable relationships
Factor means	Factor means	-	-	Cross-cultural means

Note: Comparisons are valid only if a specific model is tenable, that is, when the model for a level of measurement invariance does not fit significantly worse than the level before it. Constraints may be relaxed until a level of *partial* measurement invariance holds.

In cross-cultural research, it is often assumed that equivalence at the initial stages (factor form and loadings) is sufficient to determine whether a construct can be assessed invariantly across cultural groups [43], because levels beyond metric invariance are often difficult to achieve [40, 42, 44]. As long as one is interested only in correlations between factors this level may be fine. Yet comparing factor means (or scale means) always requires scalar (or uniqueness) invariance.

The typical approach tests for deviations from strictly identical parameters. What if the model fit does not support invariance at one level? One way out is to compose the scale of only those items that show equivalent parameters. This strategy comes at the expense of losing crucial information on specific content. A better way is to accommodate unequal parameters within the CFA framework. The notion of “partial measurement invariance” describes that equality constraints are sequentially relaxed; in other words, constraints are imposed on some but not all of the factor loadings (or intercepts; see [45]). At least two loadings (or intercepts) have to be equivalent to scale (or locate) latent variables correctly and comparably. Ideally, *most* of the parameters should be invariant on each level, while only *few* parameters differ between groups [45]. Obviously, the degree of noninvariance matters. A minority of extremely variant items may hurt cross-cultural comparisons more than a majority of items that are statistically salient but not detrimental to practical scale uses. Unequal item intercepts may either bias the factor scores or, cancel each other out, thus, hardly affecting estimated factor scores. Alternatively, Bayesian approaches to “approximate measurement invariance” can properly incorporate (negligible) nonequivalent parameters [46, 47].

Strictly speaking, if *at least* partial invariance is met, further MI tests can be carried out, including tests of structural parameters [34]. So at least *partial* metric invariance is necessary to scale the factors in each group on a common metric; and at least *partial* scalar invariance is required for numerical comparisons of latent means across cultures [36, 45]. As a caveat, the more adjustments for noninvariance are necessary below the level of uniqueness invariance, the more difficult to interpret are the residuals. Yet, even lack of measurement invariance may be informative. From a theoretical view, noninvariance can hint at different psychological processes in groups, or indicate how operationalizations should change. Yet from a practical point of view, the consequential validity of a scale may be relatively unaffected if a measurement model were misspecified such that slightly varying parameters are assumed to be equivalent.

Once (partial) measurement invariance is met, other aspects of *full* or *structural invariance* can be tested across group, such as equal factor means or factor variances and covariances. Invariance of structural parameters, a priori, are not of theoretical relevance to our research questions, yet because of the complexity of the measurement model adopted for the SBS we will later return to the structural properties, before we can safely test and establish metric invariance.

### Why Is Measurement Invariance Important in Research Involving Cultural or Self-Peer Comparisons?

The questions asked for cross-cultural comparisons may differ from those asked for self-peer comparisons, but the scientific hypotheses that can be tested always depend on the established level of measurement invariance (summarized in Table 1). Regarding *metric invariance*, as Chen [28] articulately explained, unequal factor loadings imply that the units of measurement differ, preventing a meaningful comparison of correlational relationships of a construct across cultural contexts, not to mention the implied impossibility of comparing factor means or composite scores such as scale sums or means. Unequal loadings might occur when the meaning of the concept—in this case, supernatural belief—differs across cultures, or when items have been inappropriately translated. Similarly, peer-reports can only serve as a validation criterion to the degree that peers are able to rate target characteristics along the same scale. Yet cognitive, motivational, and epistemic biases are known to affect the measurement of others’ unobserved (and often unobservable) characteristics, also, or particularly, in intimate relationships [48, 49]. For instance, targets and peers might not discuss matters of belief at all. In any dyadic situation, unequal loadings imply that peer-reports cannot be legitimately used for validating self-reports by correlation. If many items had noninvariant loadings, one might debate whether comparisons of factor means across groups are truly meaningful. If there were several items that differed strongly in their loadings across groups, this might indicate a different understanding of the construct in both groups; small deviations, however, may hardly affect any of the substantial conclusions.

Furthermore, regarding *scalar invariance*, unequal intercepts prevent clear interpretation of group differences on factor means, as (potentially valid) cultural differences are confounded with mere measurement artifacts (bias). Whenever intercepts differ across groups one cannot distinguish belief levels from different “calibrations” for expressing such belief. For instance, people from different cultures might rate their beliefs against different reference frameworks for expressing belief. Differential social desirability concerns might result in consistently higher or lower belief ratings in one group. In the context of dyads, unequal intercepts might reflect differential evaluation apprehension or modesty norms that may not apply to informant-ratings on others’ religious beliefs. Also lack of insight about specific beliefs on the belief-holder’s side might prevent equal intercepts across self- and peer-raters. Differing intercepts raise questions about their origin: Are they due to a unique understanding of items, perhaps group-specific language use or response-styles, or do they reflect a translation issue? Common cross-cultural differences unrelated to the construct—for example, in(ter)dependent self-construal and emotional display rules—can pose threats to scalar invariance too.

With regard to *uniqueness invariance*, if items lack equal residual variances, this undermines equal reliability across cultures, likewise across self- and peer-raters. Crucially, specific manifest item scores cannot be compared then because unexplained variability introduces different amounts of error to the observed scores. The comparison of composite scores is jeopardized due to the underlying different item utilization. As DeShon [39] (p.104) has aptly put it, “error variance is not only a random process, but also the effect of unmodeled sources of systematic variance that affect measured responses.” Unequal residual variances indicate that the items, dissimilarly across groups, capture random variance and/or unique variance in the form of unmodeled influences, indicating potential model misspecification.

### Current Research and Hypotheses

Given the universal relevance of supernatural beliefs and the methodological intricacies involved in assessing them, establishing whether supernatural beliefs can be assessed validly across contexts is essential. Scores from scales that lack measurement invariance can be misleading, biasing inferences about hypotheses [28].

The present research serves as an opening step to the essential question of validity of cross-cultural supernatural beliefs. First, we present an initial test of the claim to translatability and applicability of supernatural belief as assessed by the SBS to a setting different from the seminal one. Despite scores of measures targeting religiosity and related constructs [50, 51], we simply do not know if religious belief *sensu* SBS can be measured validly in any different language at all. Without global data on the SBS being available yet, we start with a glimpse at a Croatian translation. Second, ours is also the first attempt to examine measurement invariance across self- and peer-ratings of supernatural belief. Previous dyadic (family) research, has seen precursors of measurement invariance tests for partners in couple relationships or twins–who each reported *on themselves* (e.g., [52]; see [24] for current practices). Yet, across all psychological domains, self-other agreement has been estimated without establishing measurement invariance across these two sources of data. We fill this gap by showing that, like other dyadic cases, ratings of identical targets involve the non-independence of data that must be accounted for whenever distinguishable self- and informant-ratings, nested within targets, are compared.

As a test case, we contrast Croatia, a country in the Northern Hemisphere, to New Zealand in the Southern Hemisphere, where the SBS originated. Both have a similar number of residents and can be considered–in many regards–“Western” countries. Like New Zealand, Croatia has a Christian cultural heritage, but, unlike New Zealand, it has resisted secularization. Indeed, census data indicate that whereas about 42% of New Zealanders identify as nonreligious [53], 86% of Croatians still identify as Roman Catholic [54]. Furthermore, ever since its declaration of independence and the dissolution of former Yugoslavia, the country has held and maintained strong ties with the Vatican, making religious questions a matter national identity; being Croatian is in some segments of the population often equated with being Catholic. Thus, while the move from the New Zealand context to the Croatian one may not seem to be an extreme one, it in fact provides a fair test of the SBS’s robustness to translation and a first cross-cultural application (for related challenges in personality assessment, cf. [55]). Finally, only few studies on the Croatian population used supernatural/paranormal belief measures [56], and there has been no rigorous attempt at ensuring the cross-cultural equivalence of any of these tests.

A second goal of the current study is to ascertain the validity of “religious belief” as a cognitive construct via both self- and peer-reports. Although self-insight is often considered uniquely human, it is an imperfect skill, subject to various sources of bias. As Podsakoff and colleagues [57] documented, response style, need for consistency, implicit theories, social desirability, and positive affect can all distort self-reports. These biases have implications for evaluations of the convergent validity of any self-report measure, insofar as criteria are also based on self-report [58]. It may appear as though the SBS enjoys high convergent validity, as SBS scores are strongly correlated with other self-report measures of religiosity (e.g., identity, behavior), but these correlations may be over-inflated by same-source biases introduced by self-presentation and introspective limits. Given the rather complex SBS-measurement model and its facetted representation of supernatural beliefs, applying the measurement model to peer-reports may not result in good model fit empirically.

The problem of common method variance has long been recognized as a threat to validity [59, 60]. One method of mitigating method-related bias is to supplement self-ratings with a different “method” such as peer-reports [61]. However, the accuracy—that is, the predictive validity—of peer-reports are themselves contingent upon various factors, including the observability of the dimension to be rated [62–64], and the closeness of the peer being reported on [65, 66]. To establish a valid peer-version of the SBS, we therefore asked participants’ self-nominated significant others for their evaluations of ratees’ beliefs. The chosen peers were meant to be people who knew the target participant very well, alleviating the aforementioned problems of *access* to information. We then tested whether the measurement model originally developed for self-reports could recover the covariance structure and explain the underlying nature of data from knowledgeable informants. Failure to do so would *prima facie* rule out peer-reports as a suitable validation criterion for religious cognition. If measurement invariance between self- and peer-ratings can be established, we can interpret the correlations between self- and peer-ratings as further indications of convergent validity of the SBS. Despite various biases, we were not pessimistic about establishing invariance, given that peers were from the same culture and could have easily been selected as the target persons reporting on themselves.

The present study tests six hypotheses. As previous analyses on two independent samples from New Zealand converged on two specific models as the best explanation of the SBS item covariance matrix, we expect that the same models will also fit Croatian data better than competing models (Hypothesis 1). We expect that the Croatian and New Zealand SBS show at least metric, if not scalar, and, ideally, uniqueness invariance [67, 68] (Hypothesis 2). Similarly, we expect that the New Zealand measurement model will fit the Croatian SBS peer-ratings (Hypothesis 3), and that Croatian SBS will show at least metric, if not scalar, and, ideally, uniqueness invariance across peer- and self-ratings (Hypothesis 4). Assuming sufficient invariance between self- and peer-ratings, we expect that zero-order and latent level correlations between self- and peer-ratings will indicate substantial convergent validity of the Croatian SBS (Hypothesis 5). Finally, assuming validity, manifest SBS scores will predict religious behaviors such as frequency of private prayer, public attendance of religious services, and participating in religious rituals (Hypothesis 6).

## Materials and Methods

### Ethics Statement

The ethical principles as laid out by the WMA Declaration of Helsinki (2013), binding for medical research, were observed. Legally, no formal ethics approval is required for social science research in many European countries, unless the research objectives involve issues regulated by law, which was not the case (e.g., use of medications, medical devices, psychological intervention, or deception). Prior to commencement, we obtained a waiver of permission from the Croatian research institute's ethics committee.

Our procedures were in accordance with the standards published by the German Society for Psychology, an adaptation of the APA’s ethical principles and code of conduct. Participants were recruited at the Croatian colleague‘s research institution. Participants were invited and informed by a researcher (or assistant) which procedures were involved in the entirely voluntary study. Participants were assured of their anonymity. Questionnaires were handed out together with an information sheet for written informed consent. Participants who were willing to contribute simply returned the package completed.

### Croatian Supernatural Belief Scale

The ten-item SBS was translated from English into Croatian by three researchers (graduate and PhD level) at a public university. All keywords like god, demon, angel, devil, heaven, hell, miracles and souls exist in Croatian and are used in the same context as are in English. A few items were discussed to choose the Croatian wording that best represented the meaning of the original statements. We ensured the quality of the translation by acquiring a retranslation from a fourth colleague (PhD level psychology student) proficient in English and Croatian. As the sentences are straightforward, the Croatian translations of all the items were closely back-translated within minor semantic nuances. Agreement with the ten statements is expressed on 9-point Likert scales, anchored at “strongly disagree” (−4) and “strongly agree” (4).

### Participants and Procedure

This study was run as part of a larger study on individual difference variables; the other measures in the study were unrelated to the present investigation. Volunteers were recruited at a large public university, so that 642 Croatian students—from freshmen to senior year, 69.0% females, 29.6% males, 1.4% unspecified (*M*_age_ = 20.38 [18–50] years, *SD* = 2.66)—participated in the study with the permission of the faculty and the professors of selected classes. Some of the professors offered extra course credits as a compensation, but we did not follow up on which students were compensated. Study majors included Psychology, Sociology, Communication Studies, Journalism, Electrical Engineering, and Computer science. Participants returned the distributed questionnaires within one week. Five participants not providing any SBS data are not included in SBS self-report analyses.

Participants first answered a sociodemographic questionnaire, including the question “What is your religious denomination?” The majority of the university sample consisted of Christians (66.8%), followed by atheists and agnostics (29.1%), and then participants from other religious traditions (2.0%; e.g., Buddhist); 2.0% of participants did not answer the question.

Next participants completed the SBS, along with frequency measures of religious behavior, in particular how frequently they prayed, attended church/holy mass, and took communion. Table 2 displays the frequency of the religious activities as a function of religious domination. More than 95% of the Christians reported that they prayed; more than 84% claimed to go to Church at least once a year; 63% reported taking communion at least once a year.

**Table 2:** Frequency of Croatian Participants’ Religious Behavior as a Function of Self-Reported Religion.

	Never	Less than once a year	At least once a year	At least once a month	At least once a week	Every day	TOTAL
*Attends Church/Holy Mass*							
Christian	19	47	160	94	104	2	426
Atheist/Agnostic	99	57	26	1	1	0	184
Other	9	2	1	0	0	0	12
*Takes Holy Communion*							
Christian	56	102	174	63	33	1	429
Atheist/Agnostic	138	41	8	0	0	0	187
Other	11	2	0	0	0	0	13
*Prays*							
Christian	19	24	47	86	79	171	426
Atheist/Agnostic	115	31	27	5	5	3	186
Other	2	0	2	1	1	7	13

Note: Due to item non-response, *N*s = 622, 629, and 625 for Holy Mass, Holy Communion, and Prayer, respectively.

Each participant was also asked to recruit the person who knew them best to independently fill in a similar set of questionnaires about them. We stressed that the participant and the informant were to fill in the questionnaires separately and independently of each other. The completed questionnaires were to be returned one week later in sealed envelopes. Only nine participants were unable to elicit any data from their peers (these peers are not included in analyses involving peer-reports); altogether, responses from 633 peers were obtained. As part of their questionnaires, peers were asked what their relationship was to the target participants; 39.3% were friends, 23.5% were romantic partners, 19.8% were parents, and 12.8% were siblings.

Additionally, for a cross-cultural comparison, we used a previously collected sample of 360 English-speaking students to inspect measurement invariance (62.5% females, *M*_age_ = 20.92, *SD* = 3.65). These participants had been recruited at a New Zealand university and sampled from various study majors (original research presented by Jong et al. [11]). According to self-reported ethnic background (multiple nominations were possible), the majority of the sample had a European/Caucasian heritage (approx. 80%), followed by Pacific Islander, Asian, African, and Indian ethnic backgrounds. In terms of religion, 55% of the participants categorized themselves as None/Atheist/Agnostic/Undecided; 42% reported to be Christian; the rest identified as Spiritual, Free Thinker, Muslim, Hindu, Buddhist or “other”.

### Statistical Analysis and Evaluation of Model Fit

*Mplus* 7.11 [69] was used to implement the CFAs, other analyses were run with *SPSS* 21. When assuming normal theory (maximum likelihood estimation; ML) for ordinal data, this choice can yield biased parameter estimates when the number of categories is very small. Given that five or more response categories yield ML estimators that are not worse than weighted least squares estimators (WLSMV; [70–72]), we opted for ML to model the nine SBS response categories.

We obtained ML estimates of the CFA parameters with robust standard errors to account for violations of multivariate normality assumptions (self-data: χ^2^(20) = 8588.83, *p* < .0001). Without robust procedures model fit indices would be biased. Mplus provides MLR for maximum likelihood with robust ‘Huber-White’ standard errors and a scaled test statistic asymptotically equivalent to the Yuan–Bentler T2* statistic [73–75] and similar to the robust Satorra–Bentler scaled χ^2^-statistic (MLM [76, 77]). When conducting χ^2^-difference tests (or Likelihood Ratio Tests; [78]), the procedure has to be corrected for the scaling factors of robust MLR procedures [79–81]. Then the “Satorra–Bentler scaled chi-quared tests”–and other goodness-of-fit statistics based on scaled chi-square–are robust to nonnormality.

Given the limitations of any individual fit index, we tested the goodness-of-fit of plausible models using multiple criteria. First, to establish model fit, the χ^2^-test would ideally be non-significant [82], and the χ^2^/*df* ratio should be as low as possible, ideally at least as low as 2 [83]. Second, the comparative fit index (CFI) with values > .95/.90 indicates good/appropriate model fit, respectively [84, 85]. Third, the root mean square error of approximation (RMSEA) with values of .00–.05/.06–.08/.09–.10 indicates good/reasonable/poor model fit respectively [86]. Fourth, the standardized root mean square residual (SRMR) with values less than .05/.08 reflects good/ appropriate fit [85, 87]. Finally, we used the Akaike Information Criterion (AIC) [88] for single-group CFAs and the Bayesian Information Criterion (BIC) [89] for invariance tests with multiple-group CFAs [90]. Lower AIC values indicate a more accurate model, and similarly so for BIC, though BIC reflects the true data generating process better, as it penalizes overly complex (less parsimonious) models more strictly than AIC. Hence lower BIC values indicate a better trade-off between fit and complexity. For both AIC and BIC, differences in information criteria greater than +10 provide “strong evidence” against equal fit of the models in question [91].

All cut-offs for each of these fit indices are approximations and subject to model-complexity and population characteristics. The acceptance of a measurement model is not a binary (pass-fail) decision, nor does acceptance depend wholly on strict adherence to the cut-offs [92, 93]. Rather, the best practice in accepting a model involves the comparative testing of alternative models on the same data [94].

## Results

### Comparing New Zealand and Croatian Samples

To assess the applicability of the SBS to a new culture, two types of analysis were run. The first step concerned an inconspicuous test with CFAs run on the Croatian data to ascertain whether or not the same measurement models provided as good fit to the Croatian SBS as to the original SBS. This step not only replicates the seminal model selection procedure with an independent sample in a different culture; it also reduces the risk of starting the measurement invariance tests with a misspecified Croatian model. The second step invoked the crucial invariance tests to directly compare the two cultures.

Self-report SBS data from 637 Croatian participants were available. Missing values were negligible (0.87% of SBS cells), and were evenly spread across the SBS variables. Little’s MCAR test [95] across self- and peer-data showed that the pattern did not significantly differ from data missing completely at random, χ^2^ = 165.43, *df* = 209, *p* = .99. Missing data, handled by the Mplus default procedure “Full Information Maximum Likelihood” (FIML), are unlikely to adversely affect parameter estimates.

The mean SBS score was close to the midpoint of the 9-point rating scale, *M* = 0.15, *SD* = 2.27. All items of the SBS scale were highly correlated, *r* = .43–.90, *p*s < .001 (cf. Table 3, also for descriptives). On the basis of *n* = 631 complete SBS responders, Cronbach’s α = .95 (*CI*_95%_ = .94–.95). However, Cronbach’s α assumes unidimensionality; in the presence of secondary factors the assumptions for α here are unmet. Reliability estimates will be provided on the basis of SEM (see S2 File), once a measurement model has been accepted.

**Table 3:** Descriptives, Correlations, Item Loadings and Communalities with Supernatural Belief-Factor (Unidimensionality Assumed).

	Self		Peer		Correlations										Self		Peer	
	*M*	*SD*	*M*	*SD*	1	2	3	4	5	6	7	8	9	10	λ	*h*^2^	λ	*h*^2^
1 God	0.50	2.97	0.57	2.94	**.77**	.80	.78	.67	.62	.59	.86	.79	.72	.59	.87	.76	.88	.77
2 Devil	−0.51	2.89	−0.17	2.89	.76	**.62**	.79	.83	.58	.54	.78	.80	.66	.57	.86	.73	.87	.75
3 Angels	0.22	2.73	0.37	2.75	.75	.74	**.67**	.78	.67	.65	.78	.72	.75	.64	.87	.76	.90	.80
4 Demons	−0.77	2.72	−0.52	2.70	.61	.81	.73	**.54**	.60	.58	.68	.70	.62	.61	.76	.58	.82	.67
5 Souls	1.31	2.61	1.11	2.66	.66	.58	.65	.50	**.52**	.78	.64	.60	.62	.46	.74	.55	.74	.55
6 Spiritual Realm	1.75	2.45	1.50	2.49	.58	.54	.65	.50	.77	**.53**	.62	.59	.66	.50	.71	.51	.73	.53
7 Heaven	−0.01	2.92	0.36	2.92	.82	.75	.73	.61	.64	.58	**.70**	.91	.72	.61	.89	.79	.90	.82
8 Hell	−0.39	2.85	−0.01	2.88	.74	.76	.67	.64	.56	.51	.90	**.64**	.66	.57	.84	.70	.87	.75
9 Miracles	0.74	2.83	0.74	2.74	.74	.67	.75	.60	.64	.64	.73	.66	**.63**	.69	.84	.71	.82	.67
10 Prophecy	−1.29	2.54	−0.87	2.65	.52	.47	.56	.49	.43	.44	.52	.50	.62	**.49**	.62	.38	.70	.48

Note: Correlations below diagonal represent self-reports and above diagonal peer-reports; *N*s vary slightly depending on missing values. Self-peer convergence in bold.

#### Confirmatory Factor Analysis

**Fig 1:**
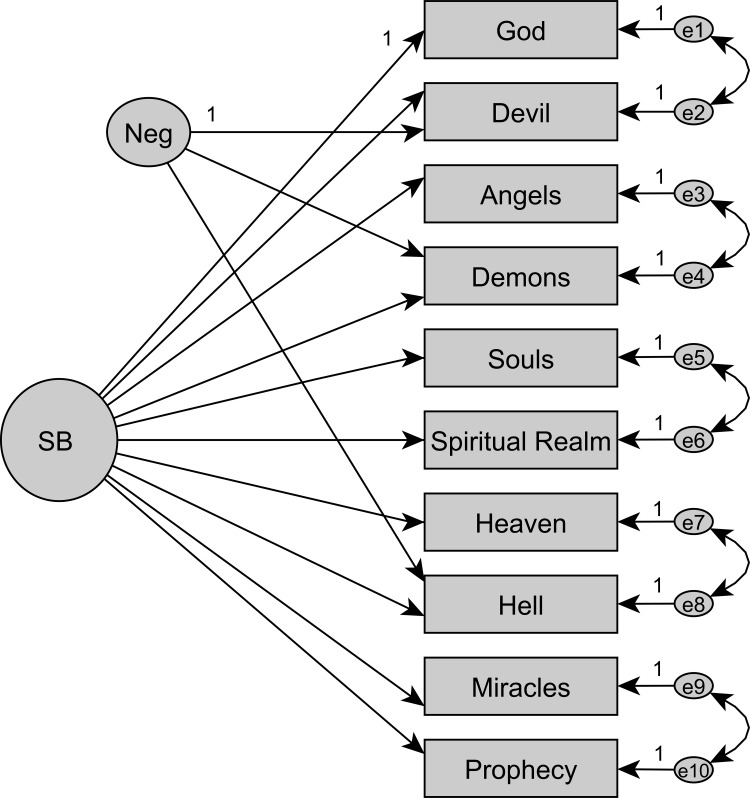
Measurement model M4. Essentially unidimensional model (SB) with method factor and five facets (correlated uniqueness) (M4).

**Fig 2:**
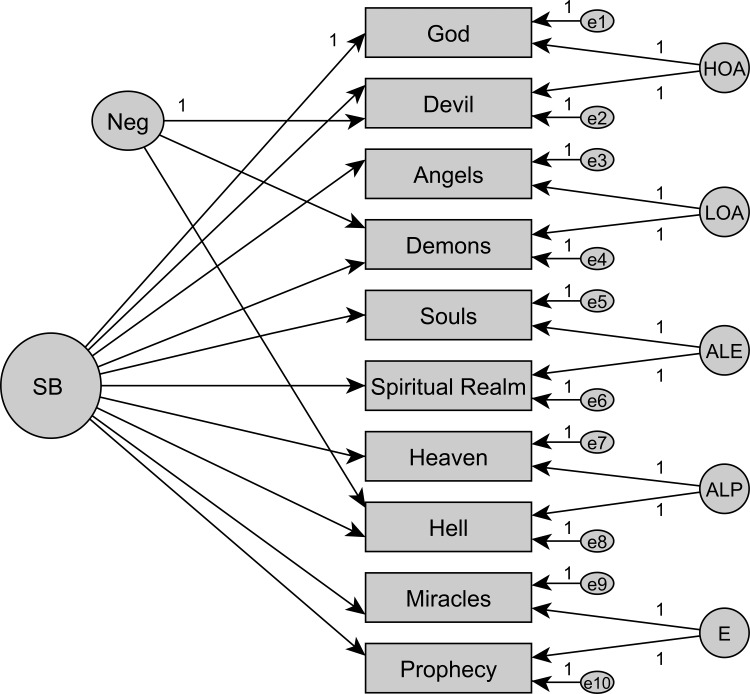
Measurement model M5. Essentially unidimensional model (SB) with method factor and five facets (content factors) (M5).

In the initial step, we used CFA to compare previously developed models (see S1–S8 Figs for models M1 to M8) to ascertain the best measurement model for the Croatian SBS (cf. S1 File). All indices converged on model fit (S2 Table). The only models to achieve sufficient model fit, supported by AIC, were essentially unidimensional models with an orthogonal method factor for negative item content and five facets for the related item-pairs (M4/M5, Figs 1 and 2). This replicates the seminal CFA results for the New Zealand sample [11]. Note that the models M4 and M5 are functionally equivalent (same degrees of freedom). By definition, there are no covariances among the factors. Yet, M5 models the item-pair specific covariance at the level of additional content factors, thereby “explaining” variance. M4 instead models the same item-pair specific covariance as if these were “unexplained” residual correlations between items. To estimate reliability, model M5 will be used.

The internal consistency of a construct is evidenced if at least half the variance can be extracted by the focal construct, AVE (average variance explained) > .50 [20]. The average variance explained in all ten indicators amounted to AVE = .81, reflecting the proportion of test variance due to all identified common factors including orthogonal method and content facets. The dominant factor by itself, that is, the common core of supernatural beliefs, explained on average 62% of the variance, reflecting the extent to which the variance of item answers can be attributed to the dominant latent supernatural belief trait. Recommended reliability cutoffs depend on the purpose and use of a scale. Estimates below .60 are generally considered too low; values around .70 may be acceptable for exploratory studies and group experiments; values around .80 are recommended for reliable interindividual differences in basic research, and .90 (with .95 desirable) is recommended for applied test use if crucial individual decisions are at stake [96, 97]. The construct reliability was high, Ω_w_ = .95, representing the maximum reliability of an optimally weighted linear combination of standardized regression coefficients of the supernatural belief factor [21]. Despite the presence of secondary factors (orthogonal sources of variance), the scale composite of a linear combination of unweighted items was reliable too, Ω = .94 [21].

Corroborating Hypothesis 1, the previously accepted model explained the data best, mirroring Jong and colleagues’ [11] findings in their New Zealand sample. Once again, the translated SBS was essentially unidimensional with all items loading on one dominant factor and with model fit significantly improved by orthogonal factors for negative content and specific supernatural content-facets.

#### Measurement Invariance Tests for Independent Groups

The crucial steps in determining whether SBS scores are comparable across groups were the step-by-step MI-tests as described above (cf. Table 1). By default, on the basis of measurement model M5, within each group the covariances among item residuals, as well as among factors, were fixed to zero, but factor variances were freely estimated across groups (model identification within each group per first item loading set to 1 for each factor). As some models failed to converge when running tests on M5, we tested invariance on the basis of M4, in which five theoretically-derived correlated item-pairs, rather than latent variables, reflect the content facets. These residual covariances were allowed to differ in the initial MI-tests, until constrained to equality in later structural invariance tests. Note that, despite the basic equivalence of the models M4 and M5, the outcomes of MI-tests could differ slightly.

A variety of fit indices were inspected, including changes in BIC. The χ^2^-difference test (or Likelihood Ratio Test; [78]), corrected for the scaling factors of robust MLR procedures [79–81], should be non-significant. As χ^2^-difference tests are notoriously sensitive to sample size despite overall good model fit [98, 99], we inspected changes in CFI and RMSEA, which should be as small as possible [100]. The use of strict cut-offs is increasingly discouraged [101, 102], but the conventional wisdom stipulates that ΔCFI ≤ −0.01 is acceptable as long as it is balanced by an increase of ΔRMSEA no greater than +0.015.

As depicted in Table 4, the configural invariance test was unproblematic. Among the conventional models, the metric invariance model clearly had the best trade-off between model fit and complexity (lowest BIC). Fixing intercepts (scalar invariance) and residual variances (uniqueness invariance) to be equal across groups decreased model fit substantially, as evident in the χ^2^-difference tests and changes in CFI and RMSEA beyond the critical cut-offs. Concerns about the appropriateness of cut-offs notwithstanding, the SBS clearly enjoys metric but not scalar invariance.

**Table 4:** Sequential Measurement Invariance Tests via Comparisons of Models with Increasing Equality Constraints: MGCFA: Croatia vs. New Zealand.

Model Comparison		Loadings	Intercepts	Residuals	Structure	*df*	χ^2^	Δ*df*	Δχ^2^	*p*	CFI	RMSEA	BIC	MI
MI1	Configural					54	165.69***	-	-	-	.979	.064	39631	Yes
MI2	Metric	**×**				65	216.39***	11	53.59	< .001	.971	.068	39617	Yes
MI3	Scalar	**×**	**×**			73	303.84***	8	107.74	< .001	.956	.080	39669	No
MI4	Uniqueness	**×**	**×**	**×**		83	318.00***	10	21.40	.018	.955	.075	39640	No
MI3a	Partial Scalar	**×**	(5&7 freed)			71	255.20***	6	45.60	< .001	.965	.072	39620	Yes
MI4a	Uniqueness	**×**	(5&7 freed)	**×**		81	270.36***	10	20.64	.024	.964	.068	39591	Yes
MI5	Variances	**×**	(5&7 freed)	**×**	Cov	88	297.98***	7	26.18	< .001	.960	.069	39601	Yes
**MI6**	**Full**	**×**	(5&7 freed)	**×**	**Cov, Means**	**90**	304.31***	**2**	**6.17**	**.046**	**.959**	**.069**	**39593**	**Yes**

Note: *N*_cross-culture_ = 360 and 637 for New Zealanders and Croatians, respectively

*** *p* < .001. Best-fitting constrained models in bold. Δχ^2^ refers to Satorra-Bentler scaled-χ^2^ difference tests.

However, the result of the omnibus χ^2^-test of scalar invariance is not evidence that all intercepts are unequal [36]. So we tested for *partial* scalar invariance across cultures [103]. On the basis of modification indices, we gradually allowed a few select parameters to differ freely across groups [45]. Partial invariance tests are considered to be exploratory and lack any strictly binding rules. If possible, only a few plausible parameters should be set free, other parameters should not shift tremendously, and the evidence for significance should be stronger than just at *p* = .05 at the minimal critical test-value of Δχ^2^ (*df* = 1) > 3.84 [36]. Some authors suggest a “5% of scale-length” heuristic to evaluate critical threshold differences [102].

Modification indices for the scalar model (MI3) suggested unequal intercepts of items #5 and #7. The groups differed how strongly they endorsed belief in “heaven” and “soul”. Participants from New Zealand had previously reported stronger belief in heaven than participants from Croatia (unstandardized intercept *a* = 0.07 vs. −0.38, both *SEs* = 0.15, ModIndex = 20.00, Δχ^2^ = 21.04). Croatians, on the other hand, endorsed the belief in souls more strongly than had New Zealanders (*a* = 1.01 vs 0.43, *SE* = 0.12 vs. 0.13, ModIndex = 25.25, Δχ^2^ = 27.60). The item bias minimally exceeded the threshold of the 5%-heuristic (0.45 for the 9-point scale). This either suggests that respondents endorsed these beliefs differently, the translations conveyed different connotations, or that our participants were differently calibrated for other reasons [28, 104]–a more item-specific post-hoc explanation for these items is difficult. The bias appears to be not too severe. Given the similar size and opposite directions of the threshold shifts, they tend to cancel each other out at the scale level. Sequentially relaxing these intercepts in the model clearly improved fit (MI3a). Hence, the SBS enjoys partial scalar invariance, warranting further invariance tests beyond this level.

With these relaxed assumptions we examined the equality of item residuals between groups (MI4a). This uniqueness invariance model fitted the data well. As the error variances of SBS items were comparable across groups, equal reliability of observed SBS scores may be assumed across groups if—in addition to equal factor loadings—factor variances are also equal, as tested next on the structural invariance level [105].

Structural invariance tests are helpful when the evaluation of structural parameters—factor means and (co)variances—is of interest. Due to the complexity of the SBS measurement model, five pairwise covariances form an integral part of the SBS measurement model under test; in the alternative model they would be reflected in additional factor loadings of five latent variables reflecting the content facets. Before we can safely assume metric invariance within the context of M4, we need to ascertain that the five pairwise covariances are also equivalent across groups. Technically, such residual covariances are mostly not considered structural parameters, but treated as error; as such, one would not expect (and test) their equivalence across groups. Yet in the case of M4, they reflect an invariant and intended part of the measurement model. Though they had been freely estimated up to here, the SBS measurement model M4 requires testing their equality across groups. This test replaces the test of equal factor loadings of the five content factors in psychometric model M5. The next model test occurred within the context of two noninvariant intercepts (MI5). It simultaneously invoked a test of equal factor variances (for both groups fixed to 1), a prerequisite to conclude to equal measurement reliability and similar impact of the error covariances on the observed scores. As from the beginning, factor covariances in both groups were fixed to zero (supernatural belief factor and method factor for negative items are orthogonal). A slight decrease in model fit, but one well within conventionally accepted limits, occurred. The prior conclusion of metric invariance was justified, although the loadings of content facets could not be tested in the metric invariance model (MI2) on the basis of M4. In sum, we confirmed Hypothesis 2 on measurement invariance.

Within the previous model (MI5), another structural property, latent SBS factor means, can be examined for group differences (the reference group’s factor mean was fixed to zero, the other freely estimated). The factor mean difference of 0.13 in standardized units (*SE* = 0.07; *p* = .06) agreed with the observed scale mean difference: Croatians had reported negligibly higher supernatural belief than New Zealanders, *M*s = 0.15 vs. −0.15, *SD*s = 2.27 vs. 2.28, *t*(995) = 2.00, *p* = .046, Cohen’s *d* = 0.13. The minimal disagreement in *p*-values between the two approaches shows that even a few noninvariant items might alter a researcher’s conclusion. Especially in the vicinity of conventional significance levels, we recommend not exclusively relying on manifest sum scores or scale means as indicators of supernatural belief levels, rather to use properly specified models in SEM. The final constraint of equal latent means (MI6) made almost no difference with regard to model fit.

We next discuss our invariance findings and derive recommendations for SBS scale use. “Many studies examining MI of survey scales have shown that the MI assumption is very hard to meet. In particular, strict forms of MI rarely hold”[106] (p.1064). By contrast, the SBS was invariant at the metric level and partially invariant at the level of scalar invariance. In the end, except for two noninvariant intercepts that did not introduce strong bias on the total scale, the SBS exhibited full measurement invariance across the two cultures. The implication is that supernatural belief, despite its faceted nature, has basically the same psychometric properties across these two cultural contexts. This is consistent with the idea that the SBS items are interpreted in a similar manner across our participant groups, and the same construct is being measured. As a consequence, belief levels (factor means) and relationships between supernatural belief and further constructs (*latent* correlations) within one culture can be legitimately compared to factor means and latent correlations in the other culture. Furthermore, the assessment of supernatural belief was equally reliable in both cultures, so that even *manifest* correlation coefficients at the level of *observed* SBS composite scores may be compared. Finally, within margins, even observed mean scores *within* each culture may be legitimately a compared *across* the two cultures. The high quality of the SBS allows researchers to compare correlations, unstandardized regression coefficients, and standardized effect sizes from experiments across the two cultures under consideration.

Our results also hint at minor cross-cultural limits of the SBS. Most but not all of the items enjoyed scalar invariance [45]. We caution readers that, when working with scale means outside of SEM, any unequal intercepts may distort the comparison across cultural groups [30]. Though this concern appears to be rather negligible for New Zealanders and Croatians, we suggest accommodating noninvariance within SEM and only then comparing the properly estimated latent factor means. Researchers should be hesitant to compare factor means across cultures, especially if intercept differences are not only statistically significant but tangible; it indicates that items are not similarly difficult for all respondents, so the construct might be differently understood. As Chen [28] noted: “As the proportion of noninvariant items increases, confidence decreases about the validity of this approach. Even when only a small proportion of the items are different, the following questions remain: Why are those items different? Is it due to specific samples or due to the scale?” (p. 1015).

In the present case, it might be that Croatians truly differ slightly from New Zealanders in accepting “souls” and “heaven” into their supernatural thinking. Alternatively, the item bias might represent a methodological artifact for unknown reasons, which affects how factor scores are estimated for each sample. Note that the two noninvariant intercepts, which were specified post hoc in exploratory fashion, represent reliable findings, as they replicated in the second Croatian data set (to which we turn next when evaluating peer-responses). The implications are, first, that direct comparisons of manifest scores of noninvariant items are discouraged; and second, that SBS aggregate scores were nonetheless hardly affected, presumably due to the small size of nonequivalence, the opposing directions of threshold shifts, and the small relative weight in the computation of scale aggregates. It is unlikely that these specific intercepts will be affected again in future cross-cultural studies.

Future research will expand on the cross-cultural robustness of the SBS beyond the comparison of Croatia and New Zealand. More importantly cross-*religious* research may discover stronger group differences when involving non-Western or non-Abrahamic religious contexts. In our sample, the average supernatural belief tendency in Croatia was practically of the same magnitude as the one in New Zealand. When comparing countries with different religious histories and societal structures (say, India or Japan), it is absolutely likely that different belief levels will emerge. Whereas from the present cross-cultural comparison we tentatively conclude that applying the SBS across cultures with similar religious backgrounds should be mostly unproblematic, other and more adjustments to the psychometric parameters may be necessary as the cultural backgrounds get more dissimilar.

### Comparing Self- and Peer-Ratings

Establishing convergent validity requires two independent assessments of the same trait ([59]). Peer-ratings are typically taken to provide such an independent source of evidence for an instrument’s convergent validity, whereas other self-report measures typically yield proxies for convergent validity conflated by same-source errors. So far, most researchers have taken the equivalence of any self- and peer-derived scores for granted. However, before interpreting any correlations between self- and peer-ratings as evidence of convergent validity, one must ascertain the comparability of the self- and peer-versions of the measurement device, in our case the SBS, especially if the construct under scrutiny is based on unobserved cognitive content, rather than on directly observable behavior.

We first ran CFAs to ascertain whether M4/M5 was the best model to accurately recover Croatian peer-ratings, just as it did for self-reports. We then ran measurement invariance tests, constraining an increasing number of model parameters to equality across self- and peer-ratings. Finally, we examined zero-order and factor correlations between self- and peer-ratings, as additional evidence for the convergent validity of supernatural belief as a construct and the SBS as a measurement device thereof.

As a preliminary analysis, we examined the extent to which peer-judgments of targets’ religious affiliation indicated reliable judgments. Peers demonstrated 89.6% accuracy in describing targets’ religious affiliation, χ^2^(4) = 673.72, *p* < .001, Cramer’s *V* = .74 (three-categorical contingency assessment). Peers classified a mere 3.3% of the self-reported Christians as atheistic/agnostic, whereas the reverse classification—judging nonreligious targets as Christians—was more likely (7.7%).

SBS reports from 633 Croatian peers were subjected to CFA. Missing values were negligible (0.28% of all cells), and were evenly spread across the SBS variables and handled by the Mplus default procedure (FIML). The mean SBS score was close to the midpoint of the 9-point rating scale, *M* = 0.31, *SD* = 2.33. All SBS items were highly correlated, *r* = .54–.91, *p*s < .001 (cf. Table 3). On the basis of 625 responders without any missing data, Cronbach’s α = .95 (*CI*_95%_ = .95–.96). Within the complex SBS structure, when the latent supernatural belief factor is optimally assessed, the construct reliability amounted to Ω_w_ = .96; the reliability of the total scale composite was Ω = .95. Yet better indictors of the reliability of manifest peer-ratings are the average variance extracted from the items by all factors, AVE = .83. For the supernatural belief factor by itself, AVE = .66.

#### Confirmatory Factor Analysis

The first analysis mirrored the analysis of SBS self-reports. All indicators converged on the quality of model M4/M5 as the most accurate model (S2 Table). Supernatural belief was confirmed as an essentially unidimensional construct that requires an orthogonal method factor for belief entities that convey negative valence plus five minor facets for specific content domains. Crucially, the measurement model developed for self-reports explains the covariance matrix observed in peer-reports very well, supporting Hypothesis 3.

#### Measurement Invariance Tests for Dependent Groups

As previously, MI-tests proceeded in four steps before we inspected structural parameters. The metric invariance test is critical because peers may hold information that is different from the targets’ and, they may weigh this information differently in their judgments about targets’ supernatural belief tendencies. Fit of the metric invariance model would confirm that targets and peers share their *understanding* of supernatural belief as a construct, including its underlying metric; this level will suffice for using peer-ratings as a convergent validity criterion for self-reports. Scalar (and uniqueness) invariance would indicate that asymmetries inherent in peers’ access to targets’ supernatural beliefs do not bias the estimation of belief levels at the level of factor means (or scale composites).

To determine whether SBS scores can be validated by peer-reports requires running MI-tests that can accommodate *nested data structures* for conceptual and statistical reasons. Unlike the comparison between independent samples from Croatia and New Zealand, the comparison between self- and peer-ratings requires taking the statistical dependency between the two raters’ judgments of the same target into account [37]. Consequently, we subjected *dependent* groups to CFA (DGCFA), accounting for correlated datasets. We thereby extend the dyadic CFA approach [24, 33], which has been applied to *dyadic data* (self and partner) in couple relationship research, where dyad members usually report on *themselves* (two individuals). Our approach also resembles the longitudinal CFA setup used for (auto-)correlated data collected across time, given that data reveal something about the *same individual* [67, 107]. Yet the present self-peer comparisons do not involve data that are nested within the same persons across time; nor do they merely involve dyads with indistinguishable members (peers, friends); but the data are dependent in a unique way because they are *nested within the same to-be-rated target* [108]. Without proper specification of this dependency, faulty model fit might be obtained, resulting in wrong conclusions about the suitability of peer-reports. The potential downside is that large and complex matrices can be prone to poor model fit and improper solutions [36].

**Fig 3:**
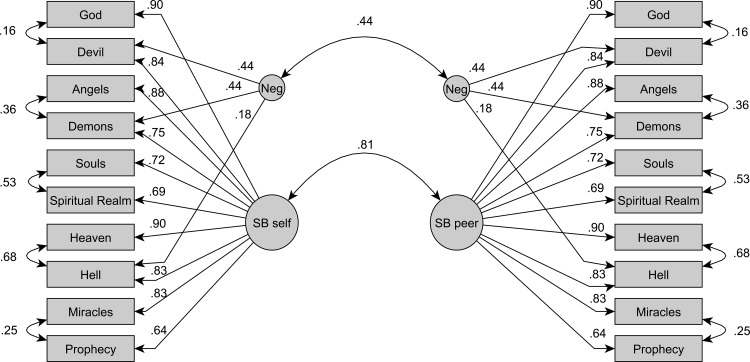
Full measurement invariance model between SBS self- and peer-reports.

We evaluated MI on the basis of psychometric model M4, given that M5 could not be identified for dependent groups. For each informant type we specified M4, including five correlated pairs of item residual variances within each group, representing the five content factors. Then we combined the two groups into one connected (dyadic) SEM: we correlated—across groups—the corresponding latent variables and the corresponding residual variances of the indicators (Fig 3). Hence, the two groups reflected dyadic assessments of the same targets by two different informants. To be clear, in addition to the self-peer correlations between the corresponding latent variables for supernatural belief and method factor, the dependency of self-peer-ratings was additionally accounted for by correlating the ten corresponding item indicators across informants (yielding ten additional covariances between the corresponding residuals). The latter paths reflect both content- and method-based covariance that cannot be disentangled further. Again, M4 models the five content facets by pairwise covariances. If M5 had been identified, there would have been five additional correlations between corresponding latent variables for the five facets. Correlations among them would have reflected and explained peers’ facet-specific knowledge about participants, while at the same time reducing the unexplained variance left to item residuals.

As a technical note, for the purpose of model identification, the loadings of the first indicator of each latent variable were fixed to 1. To override Mplus default parameters and correctly model dependent groups in DGCFA, though, all factor means in configural and metric invariance models had to be fixed to 0 (to identify mean structures), whereas they were estimated freely in the peer-group in scalar and uniqueness invariance tests.

To establish the appropriate model for configural invariance, we first checked whether modeling the covariance between corresponding item residuals across self- and peer-data was necessary at all. To do this, we compared a configural invariance model with parameters for residual self-peer covariances constrained at zero to a model with those parameters unconstrained. The (MLR-corrected) scaled-χ^2^ difference-test was statistically and substantially significant, Δχ^2^(10) = 148.02, *p* < .001. Incorporating covariances between corresponding residuals resulted in substantially better model fit than merely assuming uncorrelated residual terms; BIC, RMSEA, and CFI supported the superiority of this model. We conclude that, by not accounting for the dyadic dependency, and applying standard MGCFA to dyadic data instead, one would violate the independence assumption. Not incorporating factor correlations across informants, let alone the residual correlations, would invalidate the subsequent MI tests and bias convergent validity coefficients. By contrast, DGCFA correctly incorporates the statistical dependencies.

The model fit indices supported configural invariance (MI1, cf. Table 5). When introducing MI restrictions at each further step, model fit in terms of CFI and RMSEA hardly deteriorated. According to BIC, the restricted models at higher MI-levels rather improved in terms of the accuracy-parsimony trade-off. Thus, the self-peer comparison formally demonstrated metric (MI2), scalar (MI3), and uniqueness invariance (MI4).

**Table 5:** Sequential Measurement Invariance Tests via Comparisons of Models with Increasing Equality Constraints: DGCFA: Self- vs. Peer-Reports.

Model Comparison		Loadings	Intercepts	Residuals	Structure	*df*	χ^2^	Δ*df*	Δχ^2^	*p*	CFI	RMSEA	BIC	MI
MI1	Configural					142	460.26***	-	-	-	.966	.059	48970	Yes
MI2	Metric	**×**				153	484.19***	11	21.47	.029	.964	.058	48920	Yes
MI3	Scalar	**×**	**×**			161	528.16***	8	48.48	< .001	.960	.060	48914	Yes
MI4	Uniqueness	**×**	**×**	**×**		171	536.16***	10	12.70	.241	.961	.058	48868	Yes
MI5	Variances	**×**	**×**	**×**	Cov	178	524.32***	7	3.70	.814	.963	.055	48832	Yes
**MI6**	**Full**	**×**	**×**	**×**	**Cov, Means**	**180**	535.57***	**2**	**13.13**	**.001**	**.962**	**.055**	**48831**	**Yes**

Best-fitting constrained models in bold. Δχ^2^ refers to Satorra-Bentler scaled-χ^2^ difference tests. *N*_self-peer_ = 642

*** *p* < .001.

Recall from the cross-cultural comparison that, before metric invariance can actually be fully assumed, the psychometric model M4 for the SBS requires a test of the equality of five pairwise covariances across informants. Both the equality of variances and the equality of residual covariances are necessary within the context of this measurement model. Simultaneously fixing the factor variances of both groups to 1 and constraining the covariances to be equal across groups (MI5) did not worsen model fit, so the equivalence of relationships between the item-pairs across the dyads held (cf. Table 5).

Looking at the freely estimated SBS factor mean difference in MI5, peers’ ratings were set off by a tiny difference of standardized 0.05 scale points (*SE* = 0.03, *p* = .06). Indeed, in terms of observed scale means, on average peers significantly overestimated, albeit very weakly, how much targets themselves embraced supernatural beliefs, *M*s = 0.32 vs. 0.16, *SD*s = 2.33 vs. 2.27, *t*(627) = 2.54, *p* < .001, Cohen’s *d* = 0.07. The final test at the structural level thus concerned the equality of factor means. It requires fixing all factor means to 0 (MI6). With the exception of the notoriously sensitive Δχ^2^-difference test, no substantial decrease in model fit according to fit indices was observed (cf. Table 5). Thus, latent supernatural belief levels according to peer-reports did not systematically differ from self-reports. The full invariance model described self- and peer-data parsimoniously and accurately (cf. standardized model parameters in Fig 3), thereby supporting Hypothesis 4.

We conclude that peer-reports reflect virtually the same construct as captured by self-reports. Taking all aspects of structural invariance (e.g., equal factor variances) into account, peer-ratings have psychometric qualities (e.g., reliability) nearly identical to those of the scores elicited from the target participants themselves [105]. Consequently, manifest ratings from both groups can be legitimately compared and establish convergent validity.

#### Convergent Validity of Self- and Peer-Reports

The zero-order correlations between manifest self- and peer-reports for each of the ten SBS items ranged from *r* = .49 to .77 (*p*s < .001; cf. Table 3). The strongest consensus emerged for the belief in a high god; the weakest consensus for the belief in supernatural messengers (i.e., prophets). Aggregating to the level of SBS mean scores, both informants’ ratings converged strongly, *r*(627) = .75, *p* < .001.

However, in a scenario that is not strictly unidimensional, the theoretical nature and origin of such convergence cannot be inferred from zero-order correlations of manifest variables. Yet at the latent level of the measurement invariance model, the structural relationships reflect the correlations among the constructs of interest (Fig 3), the most important being the supernatural belief factor. In line with Hypothesis 5, the latent self-peer relationship, *r*(641) = .81, *p* < .001, reflected high self-peer agreement on individuals’ supernatural belief levels. Furthermore, the medium-sized correlation for the method factor for negative items indicated that peers had, at least, a non-trivial understanding of, if not an intimate knowledge about, targets’ specific beliefs about negatively-valenced supernatural entities. All available evidence points to high convergence between self- and peer-ratings of supernatural belief assessed with the SBS. Furthermore, that the zero-order correlation between self- and peer-SBS scores resembled the latent correlation highlights the suitability of the SBS composite score as a proxy variable to analyze belief. At the same time, the less than perfect latent correlations point to people’s limits of judging other people’s cognitions.

#### Concurrent validity of SBS scores

Evidence of concurrent validity is given by the SBS’s prediction of the frequency of three religious behaviors, namely the frequencies of praying, attending church services, and participating in the ritual of communion, respectively, Pearson-*r* = .77, .70, and .68 (*p*s < .001). Assuming more correctly that these measures were ordinal level, the rank-correlations amounted to Spearman-ρ = .75, .71, and .69 (*p*s < .001), yielding evidence for Hypothesis 6.

The benefit of using the SBS over religious self-categorization, or religious denomination, is evident from comparing the correlations computed separately for Christians and Atheists/Agnostics. Whereas a categorical response, say, “Christian” cannot differentiate between fervent and less fervent believers, the variability among Christians is reliably captured by the SBS. It capably predicted praying, attending church services, and participating in holy communion, Pearson-*r* = .60, .60, and .57 (*p*s < .001) and Spearman-ρ = .54, .60, and .59 (*p*s < .001). As the frequency of religious behaviors varied even among self-ascribed Atheists/Agnostics, their level of supernatural belief could be exploited to predict whether individuals occasionally or never showed religious activity, *r* = .53, .17, and .14 (Spearman-ρ = .52, .20, and .16; all *p*s < .05). Of course, among this group the public display of religious behavior (church attendance, holy mass) is range-restricted, limiting the correlation coefficients; yet private religious behavior (prayer) could nonetheless be inferred from SBS scores, basically as well as for Christians, *r* = .53. Thus, the SBS is the first measure capturing personal belief tendencies that predicts how likely self-declared non-religious people are to pray, although they find themselves outside of organized forms of religion. If religious people “believe what they shouldn't” [109], then certainly at least some nonreligious participants acted on grounds of what they do not yet officially believe in.

## General Discussion

### Summary

The SBS was designed as a flexible, translatable, and cross-culturally applicable measure of individuals’ tendency to believe in those supernatural entities that have been identified in anthropology as cross-culturally recurring themes. Hence, the SBS was envisioned to be used also cross-religiously. Jong and colleagues [11] provided the first evidence that the SBS was an essentially unidimensional measure that was useful for testing hypotheses about the psychological causes of supernatural belief (see also [22]). Our findings provide the first evidence for the SBS’s translatability and cross-cultural applicability.

The English and Croatian versions of the SBS enjoy full invariance, though only partially at the scalar level; yet the degree of noninvariance was almost negligible and hardly detrimental to scale means. Therefore, SBS scores are (almost) perfectly comparable across the two languages and may be used in cross-cultural replications of correlational and experimental studies. However, two specific manifest items (heaven, souls) are not directly comparable. Despite the potential usefulness of ranking supernatural belief levels across countries [110, 111], we recommend that in future cross-cultural studies researchers always take care whether manifest SBS scores–and supernatural belief in specific entities in particular–can actually be compared across nations, cultures, or languages; conclusions may be tentatively drawn on a latent level. Also, with full invariance between self- and peer-ratings, peers constitute as a valid source of information about the SBS’s convergent validity. Indeed, our findings support high convergent validity of SBS scores.

### Other Validation Attempts in the Field of Religiosity

Many scholars have conceptualized religiosity as a multi-dimensional construct [112], suggesting that affiliation, behavior, and belief form the integral components, whereas others have approached its measurement even with single-item measures [113, 114]. Apart from the underlying debate about the dimensionality of religiosity, there is no consensus about the adequate assessment of religiosity [115, 116]. Despite an abundance of scales of religiosity and related constructs, such as spirituality [50, 51], research on religiosity scales has grossly neglected the topic of cross-cultural measurement invariance. Some exceptions do exist though. Mathur [117] observed (partial) measurement invariance when comparing Indian and U.S. samples. This analysis was based on a six-item scale that claimed to reflect all three aforementioned religiosity components and nonetheless treated religiosity as a unidimensional construct [118]. Another approach to measurement invariance—on mysticism—was based on a comparison of Christian and non-Christian Chinese participants [119]. This comparison was neither focused on a religiosity measure proper, nor did the samples involve a cross-cultural comparison. Recent attempts at establishing cross-cultural invariance for spirituality have been less convincing; not even the number of factors could be reliably replicated [32]. We underscore the necessity, and at the same time the value, of inspecting cross-cultural measurement invariance of scales in research on religion. We emphasize that properly constructed and, preferably, unidimensional tests do stand a chance to be applicable in different contexts, and we expect the SBS to set a standard in this regard.

Due to the miscellaneous nature of many scales, an important development in the cognitive science of religion is to assess each religiosity component separately, reliably, and validly [19]. The SBS focuses exclusively on the cognitive factor, the belief component, and its cross-culturally recurring constituents as observed in anthropological research [11]. This scale already requires an elaborated measurement model to reflect the influence of diverse belief elements on general belief tendencies. At least among participants with different “Western” cultural backgrounds, invariance was established cross-culturally. With regard to the difference between cross-cultural and cross-religious comparisons, the latter may pose a bigger challenge than when comparing samples from countries with shared influence dominated by a, say, Christian world-view. Comparisons across heterogeneous populations (such as from India and the U.S.) or dissimilar dominant religions (say, Hinduism and Christianity) might pose challenges too. On the optimistic side, the nature of the construct was well-understood by self- and peer-raters and shared across at least two countries.

### Limitations

The current findings are not without their own challenges and limitations. First, we estimated linear relationships between manifest and latent variables in the context of nested data, but up to now these models have only been applied to longitudinal data nested within subjects, or to dyadic data nested within dyads (couples, twins), not to dyadic data nested within the targets to-be-rated (self-peer). Despite forerunners, the conceptual novelty of our approach comes at the expense of prior experience with it, especially with regard to MI-tests. Strictly speaking, CFA models are suitable for continuous variables and should not be applied to polytomous variables (4- or 5-point Likert scales) lightly. As the answering format of the SBS supplies nine graded steps, we are not concerned about the approximation by normal theory (cf. [70–72, 117]). CFA with ML estimation is relatively robust already when used with merely five ordinal response categories [120]. Given the fit of the initial measurement model in each set of self- and peer data, it appears unlikely that combining the two models into one, while adding dependencies for the dyadic structure, invalidated our attempt to establish MI. Despite a priori concerns that DGCFA might not fit well due to model complexity, there was no indication of misfit, or that dependent self- and peer-assessments differed at any MI level. By contrast, comparing two independent groups with MGCFA did detect two unequal intercepts. Dyadic data raise the power to detect differences (cf. paired and unpaired *t*-tests), so the fact that MI held for self- vs. peer-data is reassuring for the validity of this DGCFA approach.

Another concern is the possibility of “stereotypic accuracy”, a measurement artifact known to sometimes inflate correlations between people even when (and possibly especially when) they have little knowledge about each other [121]. When both raters do not describe the target, but an image of people in general, each rater will contribute to an “accurate” description in the sense that they converge on a common stereotype, inflating the inter-rater agreement. The correlation may reflect self-peer agreement that is slightly biased upward due to a holistic interpretation of how much an individual conforms to a stereotypical Croatian.

An idiosyncratic version of stereotypic accuracy is the “self-based heuristic” [122]. When peers rate targets on aspects that are difficult to observe and evaluate, they tend to fill any gaps by projecting from their own personality. As participants selected significant others who knew them well, we may have accidentally run into dyads that did not only share knowledge on ratees’ beliefs, but who shared religious affiliations, belongingness to religious communities, and supernatural beliefs too. Consequently, the convergence of self- and peer-reports may partly reflect similarity-based agreement among well-acquainted people [123]. Reassuringly, this has been a negligible concern in previous research, at least in studies on broad personality trait ratings [124]. Nevertheless, the case of the SBS is quite specific and, unlike Funder and colleagues, our study design cannot empirically determine the size of such an effect if it existed. To control for similarity-based projections future research might use round-robin designs to disentangle different source effects [66, 125].

From a methodological perspective, our design confounded cultures and languages. Any differences attributable to differences in the religious background of samples could equally likely be attributed to a language issue between the original and its translation. These aspects cannot be disentangled with the currently available samples and study design. Yet, the important message here is that the quality of translation achieved was good, and the different cultural backgrounds were not detrimental to adopting the same measurement model. Metric invariance, not to mention scalar invariance, is a requirement that too often is difficult to satisfy in cross-cultural research [45, 126]. Aside from two item intercepts, the SBS assessed a highly similar construct with equal reliability in both countries, namely the general tendency to believe in the supernatural, evident in various religions around the globe. Thus, our findings indirectly support the quality achieved for the Croatian translation.

### Outlook and Conclusion

Given measurement invariance, the underlying latent variables related to the observed item answers in like manner for Croatian self- and peer-raters, as well as for Croatians and New Zealanders. Comparing manifest item or scale means across the two cultures is possible, except for the items “souls” and “heaven” [45]. Practically speaking, the degree of noninvariance was small so that scale means approximated the information contained in latent factor means. Still, any theoretical questions should ideally be taken to the latent level. Only the full SBS measurement model captures the essence of supernatural belief free from secondary factors and accommodates different intercepts so that factor scores can be legitimately compared.

While it was not of theoretical importance for us to determine how much the supernatural belief tendencies differed across Croatia and New Zealand, future researchers may find such a comparison useful, especially when they explore religious backgrounds at the global scale, which may differ more strongly than the two cultures at hand. The measurement invariance situation may always look brighter for similar religious contexts or same-language settings. Our findings do not yet speak to the wider generalizability across religions.

Still, our results make us optimistic that many more correlational and experimental questions can be addressed with the SBS with confidence. We advise more scrutiny in the future, particularly in wider cross-cultural and cross-religious comparisons. All in all, we encourage others to use the SBS in a variety of contexts, and thus explore the utility of the SBS’s specific focus on the belief component as compared to religious commitment in a broader sense [127]. With the SBS’s help, we expect more light to be shed on the mystery of humans’ religious thinking and supernatural belief.

## Supporting Information

S1 Table Supernatural Belief Scale (Items in English). (PDF)

S2 Table CFA Model Fit: Alternative Measurement Models. (PDF)

S1 File Psychometric Models and Confirmatory Factor Analysis. (PDF)

S2 File Formulas for Reliability Computation. (PDF)

S1 Fig Unidimensional model for supernatural belief (SB). (EPS)

S2 Fig Essentially unidimensional model (SB) plus method factor. (EPS)

S3 Fig Essentially unidimensional model (SB) with five facets (correlated uniqueness). (EPS)

S4 Fig Essentially unidimensional model (SB) with method factor and five facets (correlated uniqueness). (EPS)

S5 Fig Essentially unidimensional model (SB) with method factor and five facets (content factors). (EPS)

S6 Fig Two-dimensional model (negative items and others). (EPS)

S7 Fig Two-dimensional model (agents and others). (EPS)

S8 Fig Five-dimensional model (High & low agents, after-life entities, places, events). (EPS)
