# Supplementary material for: Measuring Cross-Cultural Supernatural Beliefs with Self- and Peer-Reports
Source: PLoS One. 2016 Oct 19;11(10):e0164291. doi: 10.1371/journal.pone.0164291 (PMC5070870; doi:10.1371/journal.pone.0164291)
Supplement: S1 Table — (PDF) [file pone.0164291.s001.pdf]

S1 Table

1	There exists an all-powerful, all-knowing, loving God.
2	There exists an evil personal spiritual being, whom we might call the Devil.
3	There exist good personal spiritual beings, whom we might call angels.
4	There exist evil, personal spiritual beings, whom we might call demons.
5	Human beings have immaterial, immortal souls.
6	There is a spiritual realm besides the physical one.
7	Some people will go to Heaven when they die.
8	Some people will go to Hell when they die.
9	Miracles – divinely-caused events that have no natural explanation – can and do happen.
10	There are individuals who are messengers of God and/or can foresee the future.
