# Supplementary material for: Measuring Cross-Cultural Supernatural Beliefs with Self- and Peer-Reports
Source: PLoS One. 2016 Oct 19;11(10):e0164291. doi: 10.1371/journal.pone.0164291 (PMC5070870; doi:10.1371/journal.pone.0164291)
Supplement: S2 Table — (PDF) [file pone.0164291.s002.pdf]

S2 Table

Model type	χ^2	*df*	*p*	χ^2/df	SRMR	CFI	TLI	RMSEA	*p*-close	AIC
**Self-reports**										
Unidimensional										
M1 SB factor	626.75	35	< .001	17.91	.055	.831	.782	.163	< .001	25924
Essentially unidimensional										
M2 SB, method factor (negative items)	527.43	32	< .001	16.48	.049	.858	.801	.156	< .001	25720
M3 SB, five facets (corr. uniqueness)	299.54	30	< .001	9.98	.033	.923	.884	.119	< .001	25343
**M4 SB, method factor, five facets (corr. uniqueness)**	**101.05**	**27**	**< .001**	**3.74**	**.022**	**.979**	**.965**	**.066**	**.028**	**25055**
**M5 SB, method factor, five facets (content factors)**	**101.05**	**27**	**< .001**	**3.74**	**.022**	**.979**	**.965**	**.066**	**.028**	**25055**
Two-dimensional										
M6 Negative items, others (*r* = .92)	593.16	34	< .001	17.45	.051	.840	.788	.161	< .001	25835
M7 Agents, others (*r* = .93)	657.36	34	< .001	19.33	.067	.822	.764	.170	< .001	25855
Five-dimensional										
M8 High & low agents, after-life entities, places, events	260.30	25	< .001	10.41	.029	.933	.879	.122	< .001	25289

(S2 Table cont.'d)

**Peer-reports**											
Unidimensional											
M1	SB factor	594.85	35	< .001	17.00	.053	.841	.796	.159	< .001	25198
Essentially unidimensional											
M2	SB, method factor (negative items)	529.79	32	< .001	16.56	.050	.859	.801	.157	< .001	25039
M3	SB, five facets (corr. uniqueness)	303.90	30	< .001	10.13	.032	.922	.883	.120	< .001	24638
**M4**	**SB, method factor, five facets (corr. uniqueness)**	**145.33**	**27**	**< .001**	**5.38**	**.026**	**.966**	**.944**	**.083**	**< .001**	**24386**
**M5**	**SB, method factor, five facets (content factors)**	**145.33**	**27**	**< .001**	**5.38**	**.026**	**.966**	**.944**	**.083**	**< .001**	**24386**
Two-dimensional											
M6	Negative items, others (*r* = .95)	584.66	34	< .001	17.20	.049	.844	.793	.160	< .001	25138
M7	Agents, others (*r* = .94)	590.98	34	< .001	17.38	.062	.842	.791	.161	< .001	25106
Five-dimensional											
M8	High & low agents, after-life entities, places, events	235.24	25	< .001	9.41	.024	.940	.893	.115	< .001	24537

Note: *N*s = 637 and 633 for self- and peer-reports, respectively. Values in parentheses refer to criteria for good model fit. Best-fitting models in bold. χ^2-values are scaled χ^2-values (MLR).
