# Supplementary material for: Measuring Cross-Cultural Supernatural Beliefs with Self- and Peer-Reports
Source: PLoS One. 2016 Oct 19;11(10):e0164291. doi: 10.1371/journal.pone.0164291 (PMC5070870; doi:10.1371/journal.pone.0164291)
Supplement: S1 File — (PDF) [file pone.0164291.s003.pdf]

S1 File

To ascertain the best psychometric model for the Croatian SBS, we reinspected eight pre-specified measurement models M1-M8 originally tested in the seminal research (see S1-S8 Figures). M1 represents a strict single factor model, with supernatural belief as the single construct driving responses on all items. M2 augments M1 with an orthogonal factor to account for beliefs in negatively-valenced entities such as the devil, demons, and hell. M3 accounts for content-related item-pairs that reflect high-order agents, low-order agents, afterlife entities, afterlife places, and supernatural events respectively. M4 combines M1, M2, and M3 into a model with a dominant supernatural belief factor, negative item content, and the five content-related facets. The five content facets can also be represented by five latent variables, rather than error covariance, resulting in M5, requiring identification constraints (both items loadings from each content factor were set to unity [51]). For technical reasons, M4 and M5 yield the same model fit, yet M5 is more meaningful at the theoretical level as it explains the covariances by five latent variables rather than modeling it as unexplained residual covariance. (For the reader concerned about capitalization on chance, we also inspected a non-theoretical measurement model—with identical degrees of freedom—as a plausibility check on the impact of randomly correlated error terms; it connected first and second-half items not reflecting the hypothesized content facets. The model did not show good fit.) The remaining models test substantial content factors that are correlated (thus defining a superordinate belief factor). M6 reflects negative and non-negative items, whereas M7 reflects supernatural agents and non-agents. Finally, M8 assumes five factors, one for each item-pair that forms a content domain.

When subjecting self-reports to CFA, all fit indices converged on model fit (see S2 Table). The essentially unidimensional models with an additional method factor for negative item content and five facets for related item-pairs (M4/M5) were the only ones to achieve sufficient model fit. With M4/M5 yielding the comparatively best information values, AIC

supported the superiority of M4/M5 too. This is consistent with the CFA results for the New Zealand sample. To estimate reliability, M5 will be used.

The same findings were obtained when subjecting peer-reports to CFA. Thus, there was no disagreement between the Croatian and the seminal New Zealand analysis on which psychometric model best replicated the variance-covariance matrix of the SBS items. Also, the fact that people did not report on themselves but others did not require any adjustments of the measurement model.

Note, however, that for a proper cross-cultural comparison both groups need to be compared simultaneously in multi-group CFA; otherwise model parameters (e.g., factor loadings) might differ between groups. Likewise, the statistical equivalence of self- and peer-reports needs to be established by a CFA-approach accommodating nested data-structures.
