# Supplementary material for: Measuring Cross-Cultural Supernatural Beliefs with Self- and Peer-Reports
Source: PLoS One. 2016 Oct 19;11(10):e0164291. doi: 10.1371/journal.pone.0164291 (PMC5070870; doi:10.1371/journal.pone.0164291)
Supplement: S2 File — (PDF) [file pone.0164291.s004.pdf]

S2 File

According to Fornell and Larcker [1] (cf. [2]), the reliability of measurement is the ratio of true to observed variance, and reliability ρ of a set of congeneric items of construct η amounts to:

$$\rho_{\eta} = \frac{(\sum \lambda_i)^2}{(\sum \lambda_i)^2 + \sum Var(\varepsilon_i)}$$, with λ_i representing i = 1...k indicator loadings.

Several approaches exist that differ only with regard to what is considered to be true score or error variance, and how error variance is estimated. In the case of strict unidimensionality and uncorrelated errors, Raykov's [3] coefficient ρ is estimated as:

$$\hat{\rho} = \frac{(\sum \hat{\lambda}_i)^2}{(\sum \hat{\lambda}_i)^2 + (\sum \hat{\theta}_{ii})}$$, with $\sum \hat{\theta}_{ii} = \sum Var(\varepsilon_i)$ as fully standardized SEM parameters.

Raykov's $\hat{\rho}$ yielded the same value, $\hat{\rho}$ = .97, as an estimate with Kline's [4] formula, which is based on unstandardized parameters but weighted with the factor variance ϕ:

$$\hat{\rho} = \frac{(\sum \hat{\lambda}_i)^2 \hat{\phi}}{(\sum \hat{\lambda}_i)^2 \hat{\phi} + (\sum \hat{\theta}_{ii})}$$, with $\sum \hat{\theta}_{ii} = \sum Var(\varepsilon_i)$ as unstandardized SEM parameters.

Given that secondary factors are present in measurement model M5 so that strict unidimensionality does not hold for the SBS, part of the observed variance is actually not explained by the dominant factor, but secondary factors. Therefore, the amount of unexplained error variance entering the formulae is too low, so that $\hat{\rho}$ overestimates the reliability of the latent supernatural belief variable. (Similarly, Cronbach's alpha overestimates the reliability of the scale for measuring supernatural belief.) Instead of $\hat{\rho}$, we used omega as a preferred estimate of reliability [5] (Eq.3). The maximum reliability of the supernatural belief factor can be estimated from an optimally weighted linear combination of its k = 10 standardized loadings, which is unbiased with regard to secondary factors and their explanatory value of observed variance:

$$\Omega_w = \frac{\sum \frac{\lambda_i^2}{1-\lambda_i^2}}{1 + \sum \frac{\lambda_i^2}{1-\lambda_i^2}} .$$

The reliability of the total scale composite for measuring supernatural belief can be derived from the squared sum of standardized indicator loadings and the sum of standardized error variances, the latter being the sum of variances left unexplained by the dominant factor [5] (Eq.2):

$$\Omega = \frac{(\sum \lambda_i)^2}{(\sum \lambda_i)^2 + k - (\sum \lambda_i^2)} ,$$ with $\sum \lambda_i^2 = \sum h_i^2$ due to the first factor.

Neither Ω_w or Ω represent the amount of variance captured in the measurement in relation to the amount of variance due to measurement error. This amount of variance is represented in the index “average variance extracted” and can be calculated as:

$$\hat{\rho}_{vc(\eta)} = \frac{\sum \lambda_i^2}{\sum \lambda_i^2 + \sum Var(\varepsilon_i)} ,$$ with $\sum \lambda_i^2 = \sum h_i^2$ due to all factors.

References

1. Fornell C, Larcker DF. Evaluating structural equation models with unobservable variables and measurement error. Journal of Marketing Research. 1981;18(1):39-50. doi: 10.2307/3151312. PubMed PMID: 1981-09336-001.

2. Jöreskog KG. Statistical analysis of sets of congeneric tests. Psychometrika. 1971;36(2):109-33. doi: 10.1007/BF02291393. PubMed PMID: 1972-07992-001.

3. Raykov T. Behavioral scale reliability and measurement invariance evaluation using latent variable modeling. Behavior Therapy. 2004;35(2):299-331. doi: 10.1016/S0005-7894(04)80041-8. PubMed PMID: 2004-19190-006.

4. Kline RB. Principles and practice of structural equation modeling. 3rd ed. New York, NY: Guilford; 2011.

5. Bacon DR, Sauer PL, Young M. Composite reliability in structural equations modeling. Educational and Psychological Measurement. 1995;55(3):394-406. doi: 10.1177/0013164495055003003. PubMed PMID: 1995-39431-001.
